# Opportunities and Challenges for Machine Learning-Assisted Enzyme Engineering

**DOI:** 10.1021/acscentsci.3c01275

**Published:** 2024-02-05

**Authors:** Jason Yang, Francesca-Zhoufan Li, Frances H. Arnold

**Affiliations:** † Division of Chemistry and Chemical Engineering, California Institute of Technology, Pasadena, California 91125, United States; ‡ Division of Biology and Biological Engineering, California Institute of Technology, Pasadena, California 91125, United States

## Abstract

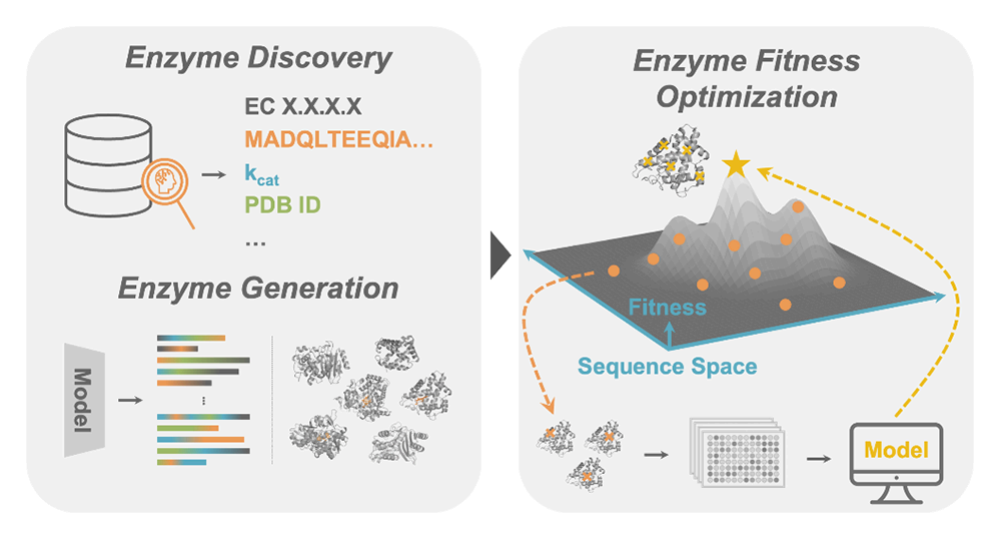

Enzymes can be engineered at the level of their amino acid sequences to optimize key properties such as expression, stability, substrate range, and catalytic efficiency—or even to unlock new catalytic activities not found in nature. Because the search space of possible proteins is vast, enzyme engineering usually involves discovering an enzyme starting point that has some level of the desired activity followed by directed evolution to improve its “fitness” for a desired application. Recently, machine learning (ML) has emerged as a powerful tool to complement this empirical process. ML models can contribute to (1) starting point discovery by functional annotation of known protein sequences or generating novel protein sequences with desired functions and (2) navigating protein fitness landscapes for fitness optimization by learning mappings between protein sequences and their associated fitness values. In this Outlook, we explain how ML complements enzyme engineering and discuss its future potential to unlock improved engineering outcomes.

## Abstract 2

Machine learning can complement enzyme engineering by helping to discover enzymes with desired function and to accelerate the optimization of enzyme fitness.

## 1 Introduction: The Current Approach to Enzyme Engineering

Engineered proteins are important for medicine, chemical manufacturing, biotechnology, energy, agriculture, consumer products, and more. Antibodies, for example, can be engineered to enhance their binding and specificity as therapeutics, whereas the stabilities and activities of enzymes can be improved under process conditions to obtain greener and more efficient chemical syntheses.^1−3^ At its core, protein engineering is a design problem: the goal is to generate and/or alter a protein’s amino acid sequence to encode a desired function. “Fitness” is a numerical quantification of that desired function, which may include multiple features that contribute to overall performance. Altering fitness is equivalent to traversing the protein’s fitness landscape, which is a surface in high-dimensional space that maps sequence to fitness. Protein engineering is challenging because accurate biophysical prediction methods for determining protein fitness are rare or nonexistent, and the search space of possible proteins is beyond-astronomically large.^4^ To make matters worse, functional proteins are scarce in the space of all protein sequences, and finding an optimal sequence on this protein fitness landscape is NP-hard, as there is no known polynomial-time solution.^5^

**Figure 1:**
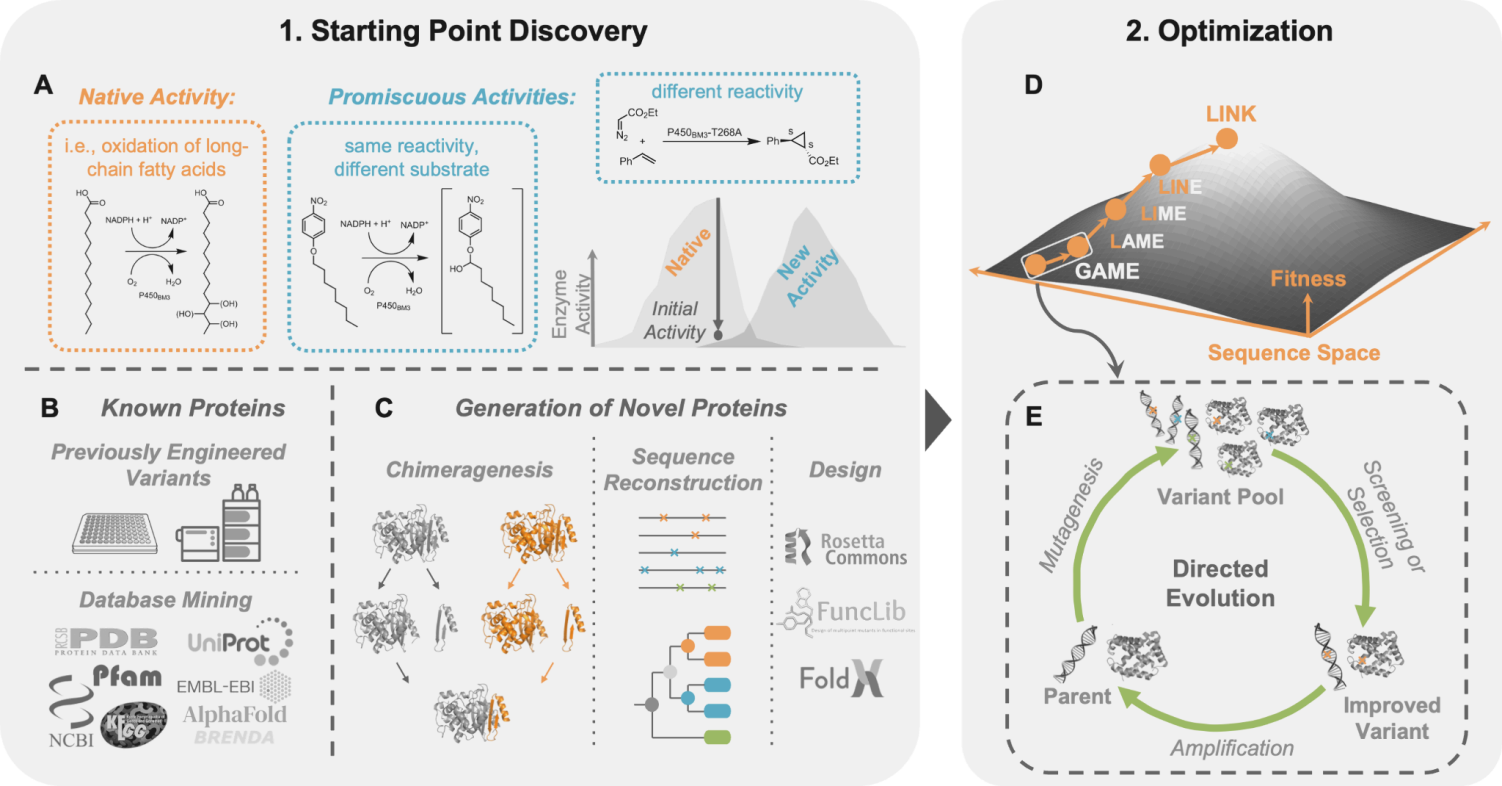
The enzyme engineering workflow. Enzyme engineering begins with a discovery phase to identify an enzyme with initial activity (desired function). If fitness is not sufficient, the enzyme is then optimized using DE. (A) Enzyme discovery involves screening for desired activities, which could include native activity or promiscuous activities. (B) Enzyme starting points can be found in known proteins or by (C) diversification of enzymes using various computational methods to generate starting sequences that are more stable and evolvable. (D, E) In its simplest form, optimization using DE involves generating a pool of protein variants, identifying one with improved fitness, and using this variant as the starting point for the next generation of mutation and screening. DE can be thought of as a greedy hill climb on a protein fitness landscape. The natural ordering of sequences in the DE fitness landscape is that all sequences are surrounded by their single mutant neighbors.^25^

In this Outlook we focus on engineering enzymes, which have applications in areas ranging from chemical synthesis and plastic degradation to diagnostics, protein therapeutics, and gene editing.^2,3^ Enzyme engineering poses some unique challenges: catalysis is more complex than binding and may involve multiple substrates, cofactors, and elementary steps. Furthermore, typical experimental screening methods for measuring enzymatic fitness are lower throughput than binding assays, for which powerful positive and negative selections can usually be devised. Enzymes are often engineered to enhance their native functions, or alternatively to target “promiscuous” activities, such as reactivity on non-native substrates or even non-native reactivities (Figure 1A).^6^ Due to the challenges of modeling catalysis and the limited throughput of meaningful assays, enzyme engineers often use directed evolution (DE) to optimize these features.^7,8^

At a high level, engineering an enzyme involves discovering an enzyme with some initial level of activity (satisfying some but not all desired properties), followed by fitness improvement using DE (Figure 1).^9^ Thus, the first step of an enzyme engineering workflow involves identifying (or designing) an enzyme with some measurable fitness. Consider engineering an enzyme to catalyze a new chemical reaction. To find a new activity that is related to a known activity, one might screen previously engineered enzymes for “promiscuous” activity for the desired function (Figure 1A).^10,11^ If none is detected, it may be necessary to explore other known enzymes or proteins in annotated databases (Figure 1B).^12^ Those with active sites amenable to accommodating a particular substrate, evolvable folds, cofactors relevant to a desired activity, or similar mechanisms may be valid starting points. Unfortunately, these approaches rely too much on experimental intuition and luck, and such an Edisonian search through existing proteins is inefficient and often ineffective. Even if activity is found, the enzyme might need to be stabilized so that it has suitable behavior for screening or can undergo further evolution, and it must express well in the host organism, such as bacteria or yeast. Computationally assisted methods such as chimeragenesis and ancestral sequence reconstruction have emerged to propose diverse protein starting points (sometimes having higher stability, evolvability, different substrate scopes) (Figure 1C).^13−15^ Methods aided by software suites such as Rosetta have been successful in redesigning enzymes and enhancing their stabilities,^16−21^ but de novo enzyme design is still nascent and works well only for relatively simple reactions.^22−24^ Because enzyme activity is influenced by a complex mix of poorly understood factors, most de novo designed enzymes must be further optimized.

Once a suitable enzyme with measurable function is identified, fitness can be improved by DE and related techniques.^7,8^ DE sidesteps the need to understand protein sequence-fitness relationships and optimizes protein fitness by performing greedy hill climbing on the protein fitness landscape (Figure 1D).^1,4,25^ In its simplest form, DE involves accumulating beneficial mutations by making mutations to the protein (mutagenesis) and screening for variant(s) with higher performance on target properties (Figure 1E). The targeted properties can change during optimization by changing the screening criteria, and informative screens can investigate multiple properties simultaneously. Recombination is often used to shuffle beneficial mutations so that screening can identify mutation combinations that further increase fitness.^26,27^ DE takes advantage of the fact that functional sequences are clustered in sequence space, i.e., functional sequences are surrounded by many other functional sequences, and smooth uphill paths exist in the landscape.^25^ However, DE is limited because screening can only explore a limited, local region within the sequence search space. Additionally, because DE largely follows a smooth path taking one mutation step at a time, so it can become stuck at a local fitness optimum.

Recently, machine learning (ML) has emerged as a useful tool for enzyme engineering, both for the discovery of functional enzymes, which is the focus of the first section of this Outlook, and for navigating protein fitness landscapes for fitness optimization, which is the focus of the second section. We encourage readers to read other reviews summarizing recent advancements in these areas.^28−37^ ML is particularly well suited for the challenges of enzyme engineering, as generative models can take advantage of patterns in known protein sequences and supervised models can learn from labels of protein properties such as various measures of fitness. In this Outlook, we explain existing methods where ML is used to assist enzyme engineering, and we propose ML-related research efforts that can have the most beneficial impact for engineering outcomes. Ultimately, we believe that the steps of ML-assisted enzyme engineering can be integrated toward fully automated engineering of many desired properties.

## 2 Discovery of Functional Enzymes with Machine Learning

**Figure 2:**
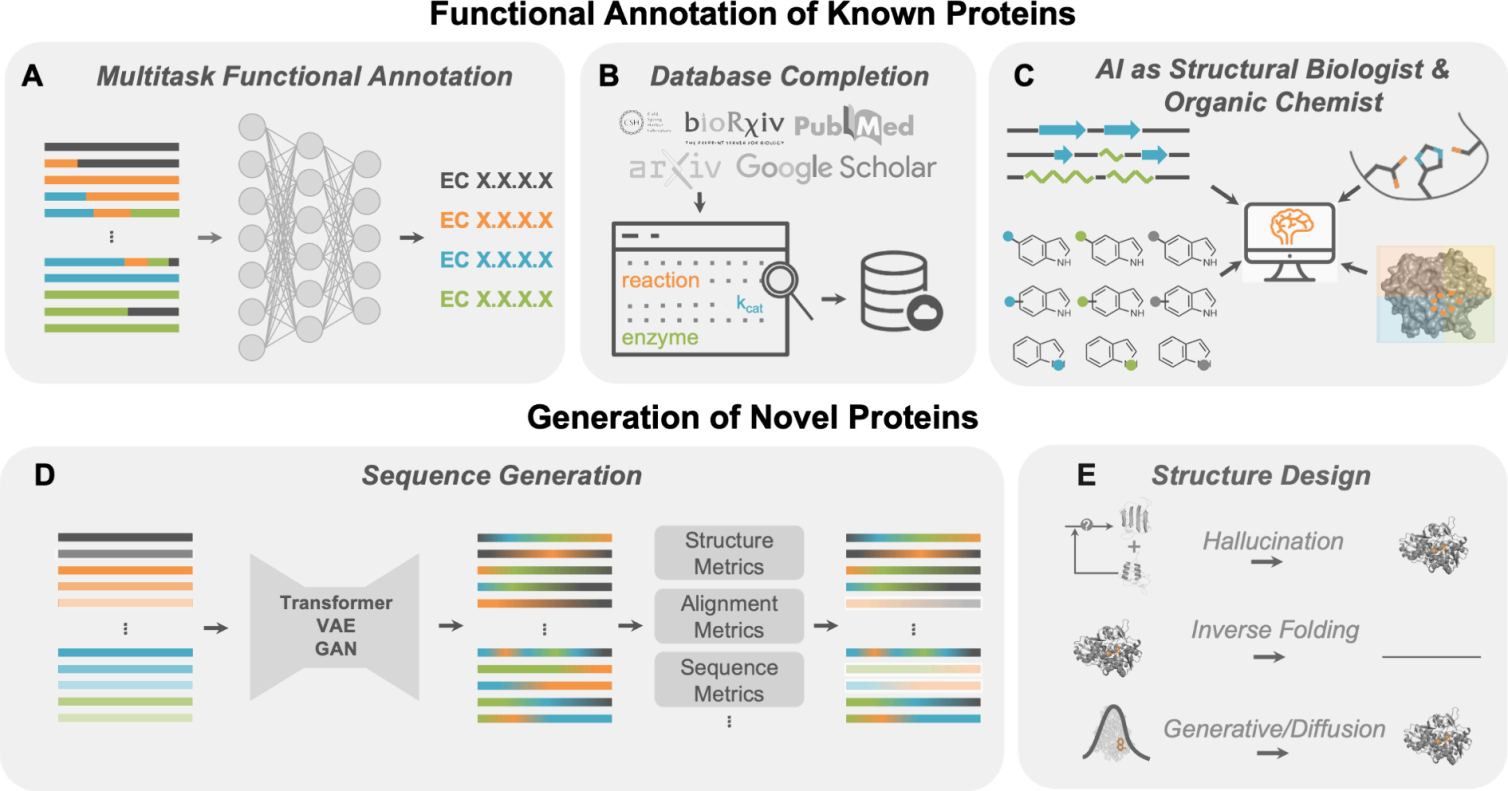
Opportunities for the discovery of functional enzymes using machine learning. Identifying functional enzymes as starting points for optimization of their properties is a key challenge in enzyme engineering. Many useful enzymes could be discovered amidst already known, but unannotated, protein sequences. (A) ML models can classify sequences based on their EC numbers. (B) Generalized LLMs could annotate proteins in databases and scientific literature, and (C) AI could act as a structural biologist and organic chemist to discern if certain reactions might work based on catalytic/structural motifs. Alternatively, emerging deep learning methods can look beyond the sequences explored by natural evolution and design novel functional enzymes. This problem can be treated as (D) pure sequence generation or (E) generation toward a target structure. Future work should focus on identifying promiscuous and evolvable enzymes.

A starting point for enzyme engineering is usually identified either from a search of existing sequences or by generating new candidates. ML methods have emerged to help with both approaches (Figure 2). Classification methods can annotate protein sequence/structure databases and uncover previously unannotated proteins with a desired function, while generative models using deep learning can design novel proteins with desired functions.

### 2.1 Annotation of Enzyme Activity among Known Proteins

Approximately 250 million protein sequences are catalogued in the UniProt database, but less than 0.3% are annotated with function.^38^ Thus, hundreds of millions of known proteins have not been explored as starting points for enzyme engineering. If these proteins could be accurately annotated, protein engineers would have access to a wealth of diverse candidates for engineering. While enzyme engineers have long been using multiple sequence alignments (MSAs) and homology to predict the functions of unannotated protein sequences,^39^ ML classification models extend these approaches and draw from more complete features describing protein sequences and structures to predict more specific functions, such as type of reactivity and *k*_cat_.^34,40−48^ Focusing on known sequences without annotations, many of these methods aim to classify enzyme sequences based on their enzyme commission (EC) numbers, which is a hierarchical classification scheme that divides enzymes into general classes and then further subclasses, based on their catalytic activities (Figure 2A).

In particular, contrastive learning-enabled enzyme annotation (CLEAN) has demonstrated state-of-the-art success at accurately classifying enzyme sequences based on their EC numbers.^40^ Upon wet-lab validation, CLEAN accurately characterized all four EC hierarchical numbers of understudied halogenase enzymes with 87% accuracy, which is significantly better than the next-best method at 40% accuracy. Impressively, CLEAN also correctly identified an enzyme with three different EC numbers, corresponding to promiscuous activities, where promiscuity prediction was framed as multitask classification.^49^ Promiscuous activities, which can include similar reactivity on new substrates or entirely different reactivity (Figure 1A), are often the starting points for evolving enzymes for non-natural activity. Thus, enzyme functional annotation efforts should include efforts to annotate these sorts of promiscuous activities for use in future enzyme discovery pipelines.^11,40^ Many promiscuous activities are difficult to detect or simply have not been tested; it will be critical to perform experimental assays to update enzyme function databases. Text mining of literature using large language models (LLMs) based on generative pretrained transformer (GPT) architectures could also help identify missing labels and update existing databases by extracting knowledge from scientific literature which has not been included in existing databases (Figure 2B).

We suggest a few other strategies to improve functional annotation efforts. EC numbers do not capture a quantitative notion of similarity between reactions, so enzyme activity prediction would benefit from a learned continuous representation of the similarity between activities, where reactions, substrates, and products are numerically encoded. This could resemble current efforts to encode chemical structures and predict the outcomes of reactions in synthetic organic chemistry.^50−53^ Databases will be useful for the curation and standardization of enzyme reaction data.^54,55^ Overall, there is also still room to develop better benchmarks for enzyme discovery, to measure the effectiveness of various models and representations.^56^

Recently, there has been an explosion in protein structure data from ML-enabled protein structure prediction tools such as AlphaFold2 and others^57−62^ and databases of unannotated protein structures. Clustering similar structures is one way to annotate for function.^63^ Alternatively, many enzymes have common “modules,” or recurring residue arrangements, which perform similar reactions.^64^ The structures of active sites in unlabeled protein structures could be compared to existing structures to identify new, diverse sets of proteins with given function, using models trained on sequence and structure.^65^ Structures could also be physically modeled to predict their interactions with different substrates. In principle, an ML model could be trained to combine multimodal information such as spatial descriptors of protein structures with an LLM trained on information about chemical reactions.^66,67^ This artificial intelligence (AI) model would act as protein structural biologist and organic chemist. By synthesizing these two forms of knowledge, the model could perform the laborious work of sifting through and identifying viable protein structures for desired reactivity (Figure 2C).^68,69^ Finally, it is also possible to go beyond known protein sequences and expand the search for functional enzymes to microbial dark matter: metagenomic analysis has only scratched the surface of these genomes.^70^

### 2.2 Generating New Proteins with Deep Learning

While many functional enzymes could be discovered through annotation of known protein sequences, generating entirely new sequences not explored by evolution could also be useful, as these could unlock unseen combinations of properties and, potentially, non-natural activities. Chimeragenesis, an approach to generating energetically favorable proteins based on recombining functional homologous proteins,^14,26^ has inspired development of deep learning approaches to assemble compatible structural domains in enzymes.^71^ Similarly, sets of mutations that are calculated to be energetically favorable using physics-based simulations (FuncLib) can be introduced in or near protein active sites to construct diversified proteins with high stability; by virtue of their sequence changes, they also exhibit promiscuous activities.^17,18,72−74^ Efforts to combine structure design methods^75−77^ and ancestral sequence reconstruction^15,75,78−80^ with data-driven models could help identify improved enzyme variants with diversified substrate scope and enhanced stability/evolvability as starting points for enzyme engineering. However, generating proteins with non-native activities will be more challenging.

While the above methods can generate diverse sequences, these sequences are still quite similar to naturally occurring sequences, which means that vast regions of protein sequence space remain underexplored. Recently, significant efforts have focused on using deep learning to design enzymes with low similarity to known sequences or structures. These efforts are reviewed elsewhere in great detail.^24,35,81−85^ In general, these methods fall into one of two main categories: (1) pure sequence generation and (2) structure design (finding a sequence that folds to a target structure or scaffold).

In pure sequence generation, protein language models (PLMs) can be conditioned by a known enzyme family to generate novel sequences with that function, without direct consideration of structure (Figure 2D).^86−98^ Models with transformer architectures have generated enzymes such as lysozymes, malate dehydrogenases, and chorismate mutases: for the best models, up to 80% of wet-lab validated sequences expressed and functioned.^88,90^ Some of these generated sequences have low sequence identity (<40%) to known proteins and may be quite different from those explored by evolution, thus potentially unlocking combinations of properties not found in nature. Variational autoencoders (VAEs) have been used to generate phenylalanine hydroxylases and luciferases, with wet-lab validation achieving 30–80% success rates.^86,87,96^ Generative adversarial networks (GANs) were also applied to the generation of malate dehydrogenases, with 24% success rate.^95^ Alternatively, a diffusion model such as EvoDiff could achieve better coverage of protein functional and structural space during generation.^99^ Despite these successes, for many methods, only a small fraction of proposed sequences are functional in the wet lab, and those that do function are often quite similar to known sequences. Simulating the structures of generated proteins, filtering them based on evolutionary likelihood, and doing other quality checks significantly increased the hit rate of functional enzymes from generative models,^100^ but there is still much room for improvement. So far, these models have been demonstrated on large enzyme families; achieving the same success on smaller enzyme families poses a challenge.

It is also possible to design desired enzyme scaffolds/structures (Figure 2E).^101−113^ One approach is hallucination, where a search algorithm uses a structure predictor to find a sequence that folds to the right structure.^103,110,35^ Luciferases with high luminescence and selectivity were engineered using deep-learning-assisted protein design, by combining hallucination with Rosetta sequence design.^107^ One of the wet-lab-validated designs demonstrated catalytic activity comparable to natural luciferases, with much higher substrate selectivity: the active site and the enzyme scaffold were both entirely different from naturally occurring luciferases. More recently, methods such as ProteinMPNN and RFdiffusion have achieved particular success for designing a broad range of proteins with targeted structures,^104,108^ where design success was validated by measuring the similarity between the target structure and the designed structure as predicted by AlphaFold2. ProteinMPNN is an inverse folding model, which is a class of models where the input to the model is a structure, and the output is a sequence. RFdiffusion is a diffusion model, where the input is a condition based on desired structure or symmetry (along with random coordinates), and the output is the coordinates of the generated structure. Still, additional wet-lab studies are needed to determine if designed enzymes can express, fold, and function.

Enzyme design still has a lot of room for growth. Designs could provide diverse starting points for further engineering of desired activities, including activities that fall outside known EC numbers. While most current success involves generating protein scaffolds or activities that are already known, it will be exciting to see more efforts that focus on generating enzymes that do not resemble those in nature and/or exhibit non-natural activities. In protein engineering, certain protein folds are more evolvable for certain reasons, including elevated stability^114,115^ that is imparted by residues outside the active site,^116,117^ balanced with flexibility to change conformation and accommodate new substrates and reactions.^118^ Proteins that express well in a host organism for evolution are also preferred. Generative models have the potential to address this need for enzymes that are better starting points than natural enzymes: for example, ProteinMPNN was able to design wet-lab validated enzymes with higher expression and thermostability.^119^ With proper labels about enzyme activity on different substrates, generative design models could be conditioned to generate enzymes with several of these desirable attributes. Future research that could address this need would be highly impactful for enzyme engineering.

## 3 Navigating Protein Fitness Landscapes Using Machine Learning

**Figure 3:**
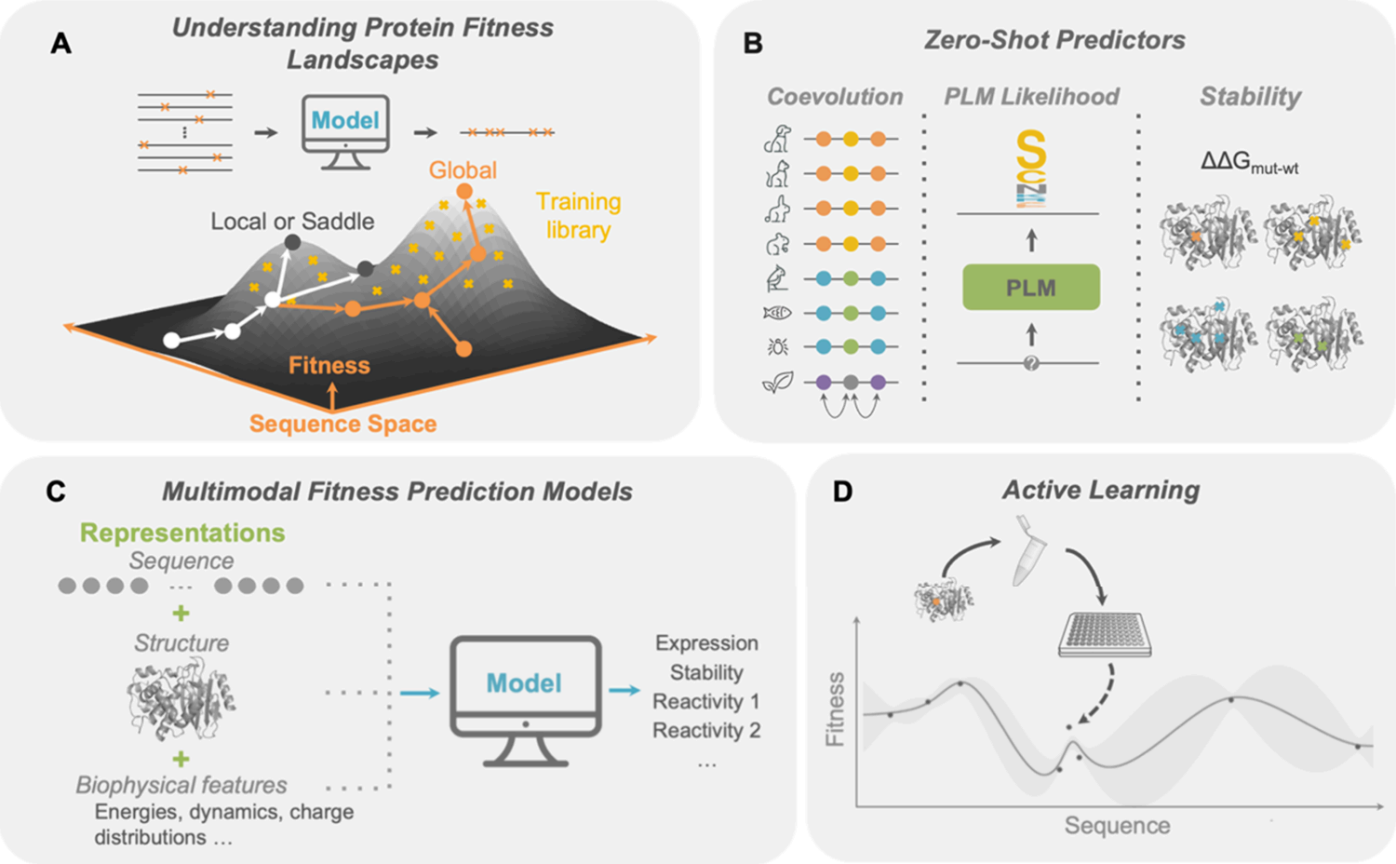
Opportunities for machine learning models to help navigate protein fitness landscapes. (A) ML models can allow for bigger jumps in sequence space by proposing combinations of mutations that would not be achieved by traditional DE. The role of nonadditivity between mutation effects, or epistasis, should be explored further to understand when ML offers an advantage. (B) The role of ZS scores to predict protein fitness without any labeled assay data needs to be better understood for different protein families and functions. Finally, ML-assisted protein fitness optimization could benefit from (C) multimodal representations that capture physically relevant descriptors of proteins to predict multiple relevant properties and (D) active learning with deep learning models tailored toward proteins and uncertainty quantification.

Most enzyme starting points identified during the discovery stage need to be further optimized to achieve desired performance levels. DE and related techniques have demonstrated success in navigating protein fitness landscapes to optimize various properties. However, DE screens or selections can sample only a small fraction of sequences in a protein fitness landscape. DE can additionally be inefficient because focusing on single mutants ignores the nonadditive effects of accumulating multiple mutations (epistasis),^120,121^ which is commonly observed when residues interact, such as in an enzyme active site or through a cofactor or substrate. Thus, a DE campaign can get stuck at a local optimum, even when high fitness sequences are nearby (Figure 3A). To address this limitation, protein fitness prediction methods using supervised ML models have emerged to learn a mapping between protein sequences and their associated fitness values to approximate protein fitness landscapes.^122−124^ These models can then predict the fitnesses of previously unseen protein variants, increasing screening efficiency by evaluating proteins in silico and expanding exploration to a greater scope of sequences compared to conventional DE approaches.^125,126^ At the same time, zero-shot (ZS) predictors—such as implicit fitness constraints learned from naturally occurring protein sequences (evolutionary conservation)—can also guide the prediction of protein fitness.^127−129^

For a protein of length N, there are ∼20^N^ possible sequences in the search space. ML models trained on the order of 10^2^–10^3^ labeled sequences (typical for an informative enzyme screen) would be unable to accurately extrapolate on such a large search space. As a result, current ML-assisted protein engineering approaches operate on constrained design spaces. Chimeragenesis has been explored as one way to constrain the search space, and various ML efforts have demonstrated success and utility on these landscapes.^122,130−133^ This approach can only introduce naturally occurring protein motifs, which can generate diverse proteins with native function while improving properties like stability. However, chimeragenesis is less likely to improve other properties, such as novel reactivity, because it retains conserved residues such as those important for native activity. More promising protein fitness prediction efforts focus on variants with one or several point mutations from a parent protein, by building training libraries using random mutagenesis^134^ or combinatorial site saturation mutagenesis. Still, artificially constraining the search space in these ways neglects certain important considerations. Using random mutagenesis to create a training library captures very limited epistasis,^135^ whereas building a meaningful combinatorial mutagenesis library requires choosing a few sites relevant to increasing fitness while still introducing epistasis, and these choices are often not obvious.

There remain many open questions about when ML-assisted protein fitness prediction is useful and how to improve it for better protein engineering outcomes, which we have summarized into the following guiding questions: (1) How should ML be used to determine the best combinations of multiple mutations on epistatic and nonepistatic protein fitness landscapes? (2) Which ZS predictors are useful in the context of native and non-native function? (3) How can ML approaches be improved to identify protein variants with high fitness more efficiently? The considerations are highlighted in Figure 3. Answering these questions is critical for advancing ML-assisted protein fitness optimization and will require new ML methods as well as new sequence-fitness data sets.

### 3.1 Combining Mutations on Epistatic and Nonepistatic Protein Fitness Landscapes

ML-assisted directed evolution (MLDE) is a specific implementation which uses supervised ML to predict the fitnesses of protein variants with multiple mutations. MLDE was demonstrated on the GB1 data set—this data set is from a combinatorial library in which four residues (with high degrees of epistasis^136,137^) were mutated simultaneously to all possible amino acids and fitness was measured by binding to an immunoglobulin protein. On this particular protein fitness landscape, MLDE was more effective than traditional protein engineering methods: it outperformed baselines such as DE using a single-step greedy walk.^138^ MLDE allowed for bigger jumps in sequence space to avoid getting stuck at local optima, which are more prevalent on highly epistatic (rugged) landscapes (Figure 3A).^129^ ML methods may be particularly beneficial where few samples are measured by assays and used for training (the *low N* regime).^133,139^ In a wet-lab validation, MLDE was used to identify a combination of mutations that resulted in an enzyme that could perform enantioselective carbon–silicon bond formation with high yield.^138^

Still, methods are needed to evaluate the prevalence of epistasis in a chosen design space to predict the utility of using MLDE over traditional approaches. As the number of simultaneously mutated residues increases, so will the epistatic complexity of the fitness landscape, and thus MLDE should be evaluated on combinatorial libraries with differing numbers of mutated sites. It is important to understand where epistatic interactions confound optimization by simple hill climbing (DE). Interacting residues near the active site of enzymes are likely to have more epistatic combinations of mutations, and the effects of mutations at these sites may be harder to predict.^140^ Similarly, studies should also explore how fitness landscapes are similar or different between different types of proteins, i.e., binding proteins, enzymes, and synthetic landscapes developed using evolutionary priors.^141^ Ultimately, combinatorial mutagenesis data sets on additional protein families are necessary for understanding when MLDE is useful. In addition to developing high-throughput assays to map protein sequences to fitnesses,^142−146^ it will be important to develop general and realistic mathematical models to describe protein fitness landscapes (Figure 3A).^141,147−150^

Alternatively, if a design space is believed to have minimal epistasis, it may be effective to assume that single mutation effects are largely additive and use recombination of beneficial mutations to find improvements. In current DE workflows, beneficial mutations found in experimental screens are mixed using methods such as DNA shuffling or StEP recombination.^7,27^ Experimental screens usually measure only a fraction of all possible single mutants, unless all sites are subjected to saturation mutagenesis, which can be time- and cost-prohibitive. Several promising studies have shown that supervised ML models can generally extrapolate well from a subset of single mutants to all possible single mutants of a protein on deep mutational scanning (DMS) landscapes, looking at natural function.^127,151^ These studies should be extended to understand how effective ML is for predicting recombination outcomes or choosing sites for further exploration.

### 3.2 Developing a Better Understanding of Zero-Shot Predictors for Different Protein Families and Functions

ZS predictors can help guide engineering toward higher protein fitness without any labeled data from experimental screens. In focused-training MLDE (ftMLDE), sampling training libraries with variants having favorable ZS scores yielded ML models with better performance than random sampling.^129^ Single mutant fitness prediction is also improved by combining sequence encodings with ZS scores,^127^ and proteins can possibly be engineered toward higher fitness using evolutionary ZS scores alone.^152^ For example, antibodies were engineered toward higher binding affinity using PLM likelihoods^128^ and higher virus neutralization using inverse folding models^153^ despite only screening 20–30 variants per round. Luciferase and chorismate mutase enzyme variants with higher stability and activity have also been identified using evolutionary ZS scores.^154−157^ The potential to improve protein engineering outcomes using ZS scores has warranted significant attention (reviewed here^158^), as calculating ZS scores does not require collecting fitness labels through expensive experimental assays. However, a method based purely on evolutionary conservation may have limitations.

Many ZS predictors have only been extensively evaluated on data sets measuring native function or activity, such as the ProteinGym DMS data sets.^151^ For example, ZS scores based on MSAs can predict protein variants that are more likely based on evolutionary conservation and coevolution.^151,159−162^ Likelihoods derived from PLMs trained on known protein sequences^88,94,151,163−171^ and inverse folding models^108,172,173^ are also able to learn these implicit evolutionary and biochemical constraints (Figure 3B). There are additional efforts to improve the accuracy of ZS predictors by using structure and reducing bias toward variants with many mutations.^174,175^ However, none of these models capture function that is not found in nature, and most studies have focused on well-studied protein families. Thus, ZS predictors need to be evaluated on proteins from different families for native and non-native functions.

Engineering enzymes for non-native activity can be challenging because many mutations that are beneficial to activity are also destabilizing.^115,176,177^ Proteins can tolerate such destabilizing mutations only up to a threshold, beyond which the protein will be unfolded.^114^ Thus, computed stability (ΔΔ*G*_mut-wt_) as a ZS score will be more correlated with fitness if the protein is marginally stable,^178^ as destabilization is more likely to cause loss of function in these proteins, such as on GB1.^129,179^ A highly stable protein, on the other hand, can tolerate multiple destabilizing mutations before it loses function; stability effects will likely not be correlated with activity for such a protein. In short, the predictive power of various ZS scores should be evaluated on existing and future data sets, to understand whether protein function, family, or other biochemical insights can be used to decide which ZS scores will be useful for a particular engineering goal.

### 3.3 Expanding the Power of ML Methods to Optimize Protein Fitness

There is also a critical need to improve supervised ML approaches to better capture patterns in data to more efficiently identify variants with high fitness. Developing higher throughput screens to obtain more data is one way to achieve improved model performance, but that of course will also improve the performance of the laboratory approach alone. In this Outlook, we focus on computational approaches that can lead to better predictions from ML models.

There is significant potential for developing more effective representations of proteins, and alongside them, evaluation metrics for these representations.^180,181^ The most simplified encodings used in ML models linking sequence to fitness include one-hot encodings of amino acid types and Georgiev parameters capturing fixed amino acid descriptors.^182^ As an alternative, learned embeddings can be extracted from PLMs, such as those mentioned above. While these representations can offer performance boosts for certain tasks,^183^ they have not yet offered significant performance boosts compared to simple sequence encodings for supervised fitness prediction in MLDE^129^ or relevant protein engineering benchmarks such as predicting multimutant fitness from the fitness effects of single mutations.^165,181^ Fine-tuning and semisupervised learning are other strategies to augment model performance when only a small amount of labeled data is available; this has shown initial promise but should be explored further.^184^ Additional benchmarks are needed to evaluate whether learned embeddings are more effective for ML-assisted protein fitness prediction.

As an alternative to PLMs, there are efforts to improve representations of proteins using multimodal data (Figure 3C). It is generally agreed that for many proteins, sequence determines structure, and structure strongly influences function. Thus, there have been efforts to enrich protein representations by incorporating structural information using voxels, contact maps, or graph neural networks.^185−192^ However, these have not led to significant performance improvements, likely because variant structures vary in subtle yet impactful ways which are challenging to model and extremely difficult to observe experimentally, despite an explosion in protein structure prediction tools. Many available protein structures may be quite noisy or inaccurate. In addition, proteins do not carry out their functions as static structures, which means that features such as dynamics and conformational changes, which could be generated using physics-based simulations or measured with experimental spectroscopic methods, could be useful.^193−198^ Because many protein fitness tasks involve variants with very few mutations from a parent protein, future efforts should explore whether representations can be learned locally on protein variants^199^ as opposed to global databases. Potentially these representations could then be fine-tuned for fitness prediction.

There has also been limited work exploring active-site focused representations,^199−201^ as the shape and electronics of an enzyme active site can strongly influence its reactivity.^202^ A related approach is taken by MutCompute, which trains a model to classify wild-type amino acids, based on their neighboring structural microenvironments.^75,76^ MutCompute was successfully used in wet-lab experiments to enhance the activity of hydrolases for PET depolymerization (plastic degradation).^77^ Joint protein–substrate representations have been studied to predict enzymatic activity for various substrate transformations, but these joint models did not perform better than independent models.^203,204^ Additionally, there exist deep learning methods that can dock substrates with proteins to predict their joint structures.^205,206^ A future generalized enzyme fitness prediction model would be able to incorporate multimodal information about both protein and substrate and simultaneously predict important properties such as expression, stability, and activity for various reactions (Figure 3C).^207^ Such models would be highly practical and impactful.

Protein fitness optimization is well suited for active learning on an expanded search space, and this area of research has significant room for growth (Figure 3D).^31,132,208,209^ Broadly, active learning is an iterative cycle that alternates between wet-lab experiments to synthesize/screen enzymes and computational modeling to propose the next set of enzymes to test, typically guided by uncertainty quantification. The goal of finding a protein variant with maximum (or at least greatly improved) fitness, is particularly aligned with Bayesian optimization (BO), which is a form of active learning. Several studies have used Gaussian process models with BO to optimize chimeric proteins.^122,130,131,133^ In an early wet-lab example, P450 enzyme thermostability was improved efficiently using an iterative BO approach.^122^ However, to engineer new enzymatic activities, protein variants with point mutations may be more interesting and promising to explore.^210−214^ BO approaches with adaptive sampling have been tested on existing data sets,^215−218^ and meta learning has been explored as way to utilize clean and noisy data for antibody engineering.^219^ An active-learning approach would more efficiently find solutions in larger design spaces, thus allowing protein engineers to expand their search to sequences with increased numbers of mutations at increased numbers of sites simultaneously mutated. An added advantage over DE is that BO allows for optimization of multiple properties simultaneously in a mathematically principled way.^220^

At the same time, new classes of ML models should be developed for protein fitness prediction to take advantage of uncertainty and introduce helpful inductive biases for the domain.^221,222^ There exist methods that take advantage of inductive biases and prior information about proteins, such as the assumption that most mutation effects are additive or incorporation of biophysical knowledge into models as priors.^223−229^ Another method biases the search toward variants with fewer mutations, which are more likely to be stable and functional.^230^ Domain-specific self-supervision has been explored by training models on codons rather than amino acid sequences.^90,231,232^ There are also efforts to utilize calibrated uncertainty about predicted fitnesses of proteins that lie out of the domain of previously screened proteins from the training set, but there is a need to expand and further test these methods in real settings.^208,233^ It is still an open question whether supervised models can extrapolate beyond their training data to predict novel proteins.^234,235^ More expressive deep learning methods, such as deep kernels,^236,237^ could be explored as an alternative to Gaussian processes for uncertainty quantification in BO. Overall, there is significant potential to improve ML-based protein fitness prediction to help guide the search toward proteins with ideal fitness.

## 4 Conclusion: Toward General, Self-Driven Protein Engineering

ML can complement many steps in existing enzyme engineering workflows, and it will play an increasingly important role in the future. Before beginning an enzyme fitness improvement campaign, classification models and generative ML models have the potential to unlock new enzymes with diverse functions, evolvabilities, and folds. Afterward, supervised ML offers a unique opportunity to accelerate protein fitness optimization by more efficiently choosing which protein variants to synthesize and screen, and it can suggest protein variants that would not normally be considered by the limited scope of DE.

On the computational side, there remain many open questions about how to use ML for enzyme engineering, and which ML-assisted methods would have the most real-world impact if successful. In this Outlook, we have suggested that discovery and generation should focus on identifying promiscuous and evolvable enzymes with new activities and folds. A wealth of diverse protein starting points remain to be discovered, and ML is well suited to identify patterns and efficiently sift through the haystack of existing proteins. ML has also demonstrated utility for navigating protein fitness landscapes, but we believe that a greater understanding of epistasis and the role of various ZS predictors is needed. Furthermore, ML models mapping sequence to fitness would benefit from improved representations of protein variants, utilization of uncertainty in predictions, and tailored models with inductive biases relevant to proteins. Here, ML allows for bigger jumps in protein sequence space than would be possible with DE. Perhaps in the future, the optimization step may not even be necessary if protein fitness information can be incorporated into generative models as part of the discovery step.

**Figure 4:**
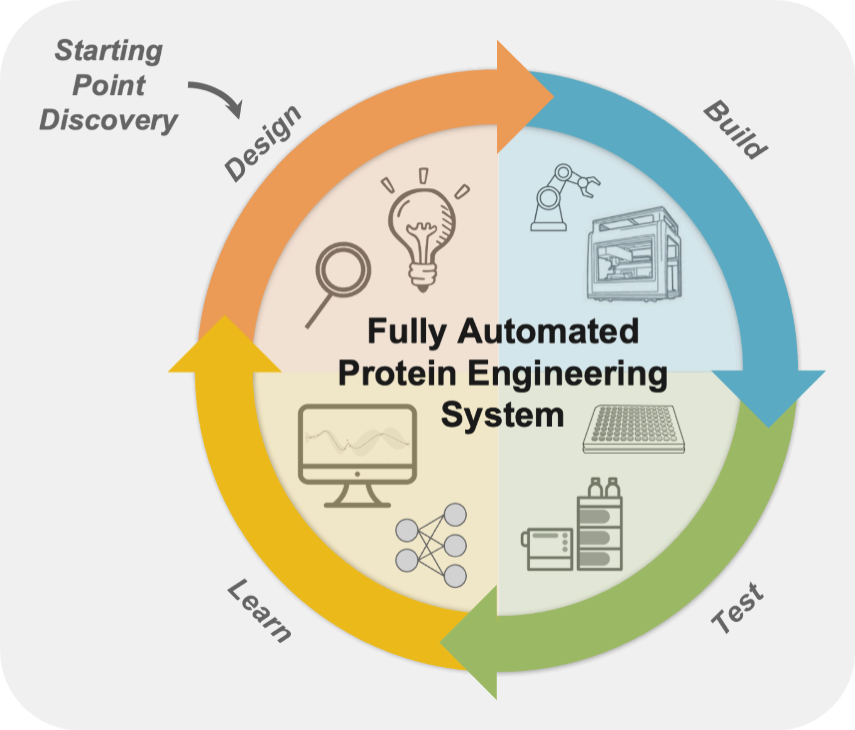
A fully self-driven protein engineering system as an active learning “design-build-test-learn” cycle assisted by machine learning. Emerging ML-assisted methods will provide an increased diversity of protein starting points that possess desired function and are highly evolvable. Automated robotic systems will synthesize protein variants and test them for various properties using experimental assays. Supervised ML models will then be trained to learn a mapping between protein features and their properties. Finally, design algorithms will propose new variants to test in the next iteration and update robotic scripts on the fly. This protein engineering system will perform automated end-to-end discovery and optimization of proteins for desired functions.

Protein fitness improvement is poised to become a fully automated process, with implications across many industries. There is already work on developing automated evolution systems and integrating these into active learning workflows where data generated from automated experiments can train and refine ML models to suggest beneficial variants to explore further.^132,238,239^ These “design-build-test-learn” cycles would enable continuous optimization of enzymes and other proteins (Figure 4), as they can for small molecules.^240^ LLMs could power these automated systems, with AI flexibly adapting to perform new types of syntheses and screens with robotic scripts written on the fly.^241−244^ At the same time, multiple desirable properties and activity for multiple reactions could be optimized simultaneously during protein engineering campaigns, powered by generalized ML models that can utilize multimodal representations of proteins. With ever increasing amounts of data on protein structures and sequence-fitness pairs, and new tools to conduct experiments^245−248^ and make ML methods for proteins more accessible to the broader community,^249^ the future of ML-assisted protein engineering is bright.

## Abbreviations

DE: Directed Evolution
ML: Machine Learning
MSA: Multiple Sequence Alignment
EC: Enzyme Commission
LLM: Large Language Model
GPT: Generative Pretrained Transformer
AI: Artificial Intelligence
PLM: Protein Language Model
VAE: Variational Autoencoder
GAN: Generative Adversarial Network
ZS: Zero-shot
MLDE: Machine Learning-Assisted Directed Evolution
DMS: Deep Mutational Scanning
BO: Bayesian Optimization
